# Navigating adulthood: Exploring the transition needs of adolescents and young adults affected by Duchenne or Becker muscular dystrophy

**DOI:** 10.1371/journal.pone.0317006

**Published:** 2025-01-14

**Authors:** Sharon Barak, Shirley Ackerman Laufer, Michal Gudinsky Elyashiv, Sharon Rubinstein-Shatz, Rama Davidson, Tali Kaplan

**Affiliations:** 1 Department of Nursing, School of Health Sciences, Ariel University, Ariel, Israel; 2 Department of Pediatric Rehabilitation, The Edmond and Lily Safra Children’s Hospital, Chaim Sheba Medical Center, Tel Hashomer, Israel; 3 Little Steps Association for Children with Duchenne Muscular Dystrophy and Becker Muscular Dystrophy, Kefar Saba, Israel; University of Miami Miller School of Medicine: University of Miami School of Medicine, UNITED STATES OF AMERICA

## Abstract

For individuals with Duchenne or Becker muscular dystrophy (DMD and BMD, respectively), transitioning to adulthood presents significant challenges. Although considerable attention has been given to facilitating medical transitions due to the complexity of these conditions, less focus has been placed on other aspects of the transition, such as achieving independence. This study assessed the transition needs of people with DMD or BMD, exploring various domains including health, education, employment, living arrangements, transportation, daily activities, and independent personal life. Men with DMD or BMD participated in this cross-sectional study. Transition to adulthood was assessed using Transition Readiness Assessment for Young Adults with DMD. The questionnaire evaluates transitions in health care, education and employment, housing and transportation, activities of daily living, and independent life and autonomy. Factors associated with and predicting transition to adulthood were evaluated using Spearman’s correlations and multiple regression analysis. Forty-two people with DMD or BMD (mean age: 24.3±5.3) participated in this study. The transition domains in which most participants needed help were education and employment (52.5%) and activities of daily living (57.0%). Transition needs that stood out included palliative care (66.6%), employment and education support (76.1%), social worker consultation for housing assistance (76.1%), and assistive device consultation (64.2%). Mobility and breathing function did not correlate with transition level. Number of siblings positively correlated with and predicted most transition domains. Older age predicted only education and employment status. In conclusion, the analysis showed that the most problematic transition domains among people with DMD or BMD were activities of daily living and education and employment. In most transition domains, help needed did not decrease with age and was not affected by function. However, adolescents and adults with more siblings typically reported being more ready to transit to adult life.

## Introduction

The transition from adolescence to adulthood is a critical phase that significantly affects the integration of young individuals into society [1]. This phase involves assuming new responsibilities and encompasses significant life events that shape individuals as they enter adulthood. The typical age range for this transition is 18 to 25 years old. Nevertheless, considering the many challenges at this stage of life, it is not uncommon for the transition phase to extend beyond age 25 [2].

For people living with high health and medical needs, the transition to adulthood is a particularly difficult task. One interesting population in the context of the transition to adult life is individuals with special health and medical needs related to Duchenne and Becker muscular dystrophy (DMD and BMD, respectively). The population of individuals with DMD holds special significance due to advancements in clinical management that have considerably extended their lifespan, transforming their adolescent years from a period marked by the end of life into a transitional phase towards adulthood [3]. Compared to DMD, individuals with BMD have a longer life expectancy and typically live well beyond their third decade. This life expectancy characteristic among BMD is an even stronger argument that proper care, guidance, and self-management for these adolescents are critical for successful transition into adulthood [4].

Considering that DMD and BMD are complex medical conditions, much attention has been focused on facilitating medical transition. For example, several important organizations, such as the American Academy of Pediatrics, American Academy of Family Physicians, and American College of Physicians [5], have well-established medical transition recommendations for young adults with special health care needs. Specifically, these organizations recommended that formal written transition plans be initiated for all adolescents starting at age 14. Despite these recommendations, only 1 in 4 male adolescents with DMD or BMD reported having a written summary to assist in the transition from a pediatric health care provider to an adult health care provider [6].

Alongside research on the transition to adulthood in the medical domain, exploration of independence in other transition domains is needed. As DMD or BMD progresses, individuals experience increasing muscle weakness and degeneration, leading to significant physical disabilities. This progression often results in reduced mobility and growing dependence on others for daily activities, such as personal care and mobility. The loss of muscle function typically necessitates assistive devices and caregiver support, diminishing physical independence. Accordingly, research has shown that individuals with less severe dystrophy, or BMD, reported higher rates than those with DMD of frequently staying home without supervision (50% vs. 14%, respectively), independently performing daily physical needs (93% vs. 7%), and having full- or part-time employment (33% vs. 4%). Most had not been in a romantic relationship but reported desiring such relationships. However, despite dependence due to physical disability, these individuals can still maintain autonomy. Autonomy involves self-determination and the ability to make life choices, which can be preserved even when physical independence is compromised. Therefore, the current study did not solely focus on an individual’s ability to perform life-related tasks independently (independence) but also examined their autonomy in managing their life [6].

Considering the special needs among people with DMD and BMD and to enhance autonomy in the transition to adulthood, education, guidance, and preparation are crucial components [7]. However, to develop new interventions to enhance and support transition to adulthood, it is crucial to understand the challenges faced by individuals with DMD or BMD in their journey to adulthood. Relatively many studies have consider the transition to adulthood among individuals with disabilities in general [8]. However, there is no current consensus or established protocol on how to carry out the transition process in an effective manner that ensures patient care and quality of life [9]. Studies are needed to assess the impact of DMD or BMD and degree of disability (e.g., motor, pulmonary) on adult role attainment and transition [6].

The objective of this study was to assess the transition to adult life among individuals with DMD or BMD. More specifically, the study examined the degree of transition (e.g., fully completed) in various domains such as health, education and employment, living arrangements and transportation, daily activities, and independent personal life. Factors related to and predicting the transition were also examined.

## Materials and method

### Study participants

This cross-sectional study featured male participants aged 16–35 and diagnosed with DMD or BMD. All families listed in a DMD and BMD database who met the inclusion criteria were contacted, regardless of their concerns regarding transition. Participant recruitment started on August 18, 2023, and ended on November 1, 2023. Exclusion criteria included cognitive or other difficulties that might hinder the ability to complete a questionnaire. Individuals with cognitive problems were excluded because the study questionnaire was not validated for individuals with such conditions, restricting our ability to recruit them. In this study, 8% of potential participants were excluded due to cognitive impairments. Among individuals with BMD, this rate aligns with the lower range of previously published studies, which reported a prevalence of intellectual disabilities between 7% and 25%. However, when compared to the DMD population, this rate is notably lower, given previous studies indicated that 17%–27% of individuals with DMD have intellectual disabilities [10]. The lower prevalence of cognitive problems observed in our study compared to previous reports may be attributed to several factors. Differences in assessment methods could play a role, with various tools used to evaluate cognitive function that may focus on specific domains or provide broader measures like intelligence quotient. Additionally, variations in genetic mutations and environmental influences, such as access to education and supportive resources, might contribute to better cognitive outcomes. Furthermore, the age and disease stage of our participants may differ from those in other studies, and cognitive impairments often become more apparent with disease progression. Given the small number of potential participants with cognitive impairments and their unique needs, this study was not designed to address the specific requirements of this subpopulation. In addition, 28% of approached families did not complete the online questionnaire and therefore, were excluded from the study.

### Procedures

This study was conducted in the Middle East (Israel). This is of special interest because most studies on the topic have occurred in Europe and the United States. In the focal country for this study, children with health conditions receive care through specialized pediatric clinics, either in hospitals or the community. Some hospitals have developed dedicated transition clinics to support adolescents as they move from pediatric to adult health care services. These clinics often focus on chronic conditions such as diabetes, cystic fibrosis, or muscular dystrophy. Transition programs typically start during adolescence, emphasizing education about their condition, fostering independence, and preparing them for adult health care settings. For youth with disabilities like DMD or BMD, organizations work with adult rehabilitation facilities to ensure seamless continuity of care. Additionally, nonprofits and advocacy groups play a vital role in facilitating and supporting these transitions.

Study participants were identified via the Little Steps Association, a nonprofit organization founded in 2010 by parents of children with DMD or BMD. Little Steps Association maintains a comprehensive database of all individuals with DMD or BMD treated in various clinics throughout the country. As such, the data in the current study had good generalizability for individuals with DMD or BMD in this country.

The research process involved contacting all eligible individuals in the database and asking them to participate. In the case of minors (younger than 18), parental permission was also obtained. The questionnaire was delivered online, with a research coordinator available to address any inquiries. Parents were permitted to provide physical assistance to participants in completing the questionnaire and if necessary, the research team offered additional support. Regarding clinical data used in this study, such data are regularly submitted to the Little Steps Association via clinics treating individuals with DMD or BMD nationwide. The data transfer process began with families signing an authorization form granting permission for the release of medical and social information. This authorization allowed medical personnel and institutions, including health maintenance organizations; their physicians, employees, and representatives; and employees of the National Insurance Institute and professionals in social or caregiving fields, to provide relevant information. The shared data included medical, social, caregiving, and rehabilitative details, along with records of past and current illnesses. This comprehensive access to information was conducted in compliance with ethical standards. An institutional review board approval for the study was obtained from [blinded for review]. All adult participants (aged 18 or older) gave their written consent to participate in the study. For participants younger than 18 years old, parents provided written informed consent.

### Outcome measures

#### Demographic information

Demographic data, such as age, living arrangements, number of siblings, and self-defined religiosity, were extracted from the database. Regarding the latter, three religiosity levels were defined: secular, traditional, and religious. Secular is a social category, designating the least religious segment". Traditional is a term used for people who identify as neither strictly religious nor secular. Religious is a term that refers to a social or religious group leading an observant lifestyle.

#### Transition level to adult life assessment

Participants’ transition level to adult life was assessed via the Toolkit for DMD. The questionnaire was adapted from the work of Trout et al. [11] and is part of the standard of care published in 2018 [12]. The toolkit is recommended by the Centers for Disease Control and Prevention and Parent Project Muscular Dystrophy, the largest and most comprehensive nonprofit organization in the United States focused on finding a cure for DMD. Accordingly, it is used in clinics treating individuals with DMD to communicate their needs.

The toolkit consists of a comprehensive checklist of items to discuss with young adults with DMD during their transition. Specifically, it consists of six domains: health care (16 items pertaining to general health and familiarization with health care providers), education and employment (nine items), housing (five items), transportation (three items), activities of daily living (four items), and independent life and autonomy (12 items). For this study, the housing and transportation domains were combined to create one domain. In each domain, answers were on a 3-point scale: “need help” (has not started or has made very limited progress in addressing the transition task or domain), “partially completed” (has started addressing the specific transition task or domain but has not fully achieved the required level of readiness), or “fully completed” (has successfully addressed the specific transition task or domain and no longer requires assistance in this area).

Responses were used both as continuous scores (1 = *need help*, 2 = *partially completed*, 3 = *fully completed*) and dichotomized scores (0 = *need help or partially completed*, 1 = *fully completed*).

#### Clinical information

Clinical data were collected to examine the impact of functionality and disease progression level on transition readiness. Ambulatory status was categorized into three levels: independent with no assistive devices, partial use of assistive devices (only for outdoor ambulation or long distances), and full use of assistive devices (for outdoor ambulation, long distances, and at home). Respiratory status was categorized into four breathing support levels: independent, nonintrusive assisted breathing for part of the day, noninvasive assisted breathing throughout the day, and fully intrusive assisted breathing.

### Statistical analyses

#### Data normality

Data normality assumptions were evaluated visually using probability and quantile-quantile plots. In addition, supplementary to the graphical assessment, a Kolmogorov-Smirnov normality test was conducted [13]. These analyses showed that the dependent measure (transition level) was not normally distributed and therefore, nonparametric statistics were used.

#### Internal reliability

The internal reliability of the questionnaire and its various parts was tested using Cronbach’s alpha. An alpha less than .50 is considered unacceptable, whereas .50–.60 is considered low, .61–.70 is disputed, .71–.80 is acceptable, .81–.90 is good, and greater than .90 is excellent [14, 15].

#### Transition level to adult life status

Transition level was examined jointly for DMD and BMD because both dystrophies have a common pathology; both are caused by mutations in the dystrophin gene, leading to muscle degeneration disorders collectively known as dystrophinopathies. Traditionally, DMD and BMD have been viewed as distinct entities, with DMD presenting more severe symptoms and earlier onset compared to the milder and later-onset BMD. However, emerging perspectives suggest that these conditions represent a continuum of clinical severity rather than separate disorders. By adopting this continuum approach, clinicians and researchers can better appreciate the nuanced differences and similarities between DMD and BMD, ultimately leading to improved diagnostic accuracy and tailored therapeutic strategies [16].

For all items, the frequency of participants who reported each stage of transition (need help or partially completed versus fully completed) was calculated. Differences in transition readiness stages were compared with chi-square tests.

#### Differences between domains in transition readiness

To compare the level of transition readiness in the six transition domains assessed, the average score of each domain was calculated and presented using a box-and-whisker diagram. Significant differences in scores were compared with Friedman’s nonparametric test.

#### Associations and prediction of different areas of transition

Associations between the different areas of transition and associations with demographic (age and number of brothers and sisters) and functional (mobility and respiratory functions) characteristics were tested using Spearman correlations. Also, differences in transition scores by type of muscular dystrophy were examined using a Mann-Whitney U test. Variables found to have a significant statistical relationship or that differed from each other in the Mann-Whitney U test were entered into a multivariate regression model to test their ability to predict the stage of transition.

All statistical analyzes were performed in SPSS software (version 19). The significance value was set at *p* < .05 (two-tailed).

## Results

### Study participant characteristics

Forty-two male participants with DMD or BMD participated in this study. For both DMD and BMD, most study participants had deletion mutation (67.9% and 75.6%, respectively), followed by duplication mutation (10.2% and 13.4%). The average age was 24.3±5.3 years (median: 23; range: 16–35). Regarding race and ethnicity, all study participants were born in the country and were of mixed race (parents or grandparents were immigrants from Europe, Arab countries, Iran, Turkey, or Central Asia). In addition, most study participants made full use of assistive devices for mobility (*n* = 28, 66.6%s; Table 1).

**Table 1:** Participant demographic, clinical, and functional characteristics (*N* = 42).

Variables			Mean (SD) [range] or n (%)
**Demographic characteristics**	Age, years: mean (SD) [range]		24.1 (5.3) [16.0–35.0]
	Siblings, number: mean (SD) [range]		3.9.0 (2.1) [1.0–9.0]
	Religion and religiousness: n (%)	Secular and Traditional Jewish	26.0 (61.9)
		Religious Jewish	12.0 (28.5)
		Not-Jewish	4.0 (9.5)
	Living arrangement: n (%)	With family	34.0 (80.9)
		Not with family	8.0 (19.0)
**Clinical and functional characteristics**	Dystrophy type: n (%)	Duchenne	25.0 (59.5)
		Becker	17.0 (40.4)
	Ambulation ability: n (%)	Full usage of assistive devices	28.0 (66.6)
		Partial usage of assistive devices	4.0 (9.5)
		Independent with no assistive devices	10.0 (23.8)
	Respiratory functions: n (%)	Full-invasive assisted breathing	2.0 (4.7)
		Non-invasive assisted breathing during the entire day	10.0 (23.8)
		Non-invasive assisted breathing not during the entire day	5.0 (11.9)
		Independent	25.0 (64.2)

Notes: SD, standard deviation.

Regarding differences between the DMD and BMD groups in main demographic, clinical, and functional characteristics, in the DMD group, 100% of participants reported using full-time mobility devices, compared to 37% in the BMD group. Regarding respiratory support, 60% of individuals with DMD were breathing independently or using partial noninvasive respiratory aids, whereas 100% of individuals with BMD could breathe independently. The mean age in the DMD group was 26.43±8.15 years; in the BMD group, it was 23.45+5.13 years (*p* = .06).

#### Internal reliability of the questionnaire

The internal reliability of the entire questionnaire was excellent (α = .96). The internal reliability of the questionnaire sections ranged from good (housing: α = .87) to excellent (all others: α ≥ .90).

#### Differences between domains in transition readiness score

**Fig 1:**
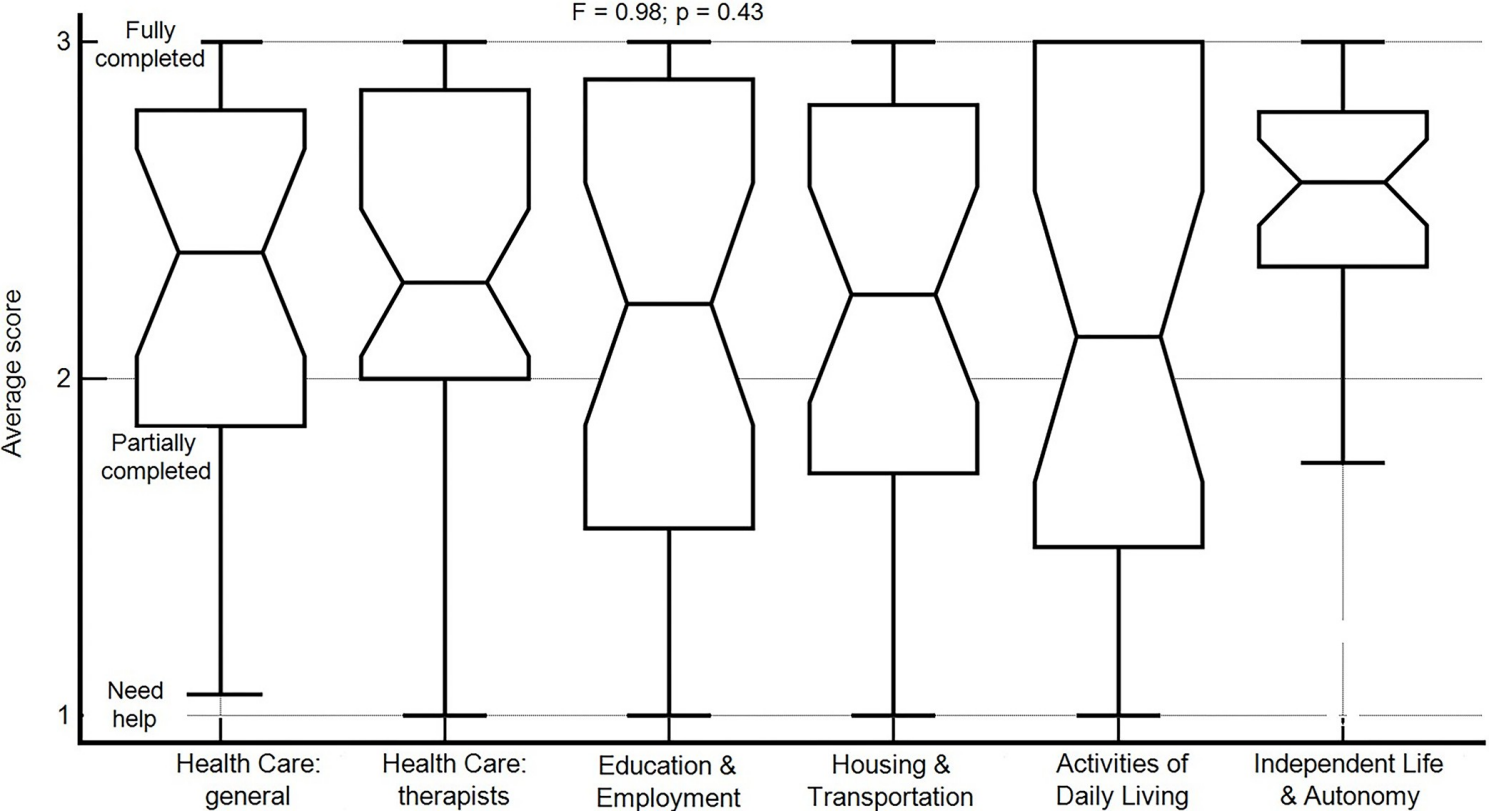
Readiness for the transition to adult life: Domains descriptive statistics. The rectangle in the center represents the values from the lower to the upper quartile (25th to 75th percentiles); the vertical line is from the lowest to the highest score; the horizontal line represents the median score.

Fig 1 indicates that the participants varied greatly in their level of readiness for transition, with median scores ranging from 2.12 (activities of daily living) to 2.58 (independent life and autonomy). No statistically significant differences were found in the average score of the various assessment areas (*F* = 0.98, *p* = .43).

#### Transition readiness status by domains and items

The survey included five assessment domains. Tables 2 to 5 show a breakdown of the proportion of participants who reported they need help or partially completed versus fully completed each item in the various domains.

**Table 2:** Transition readiness–health care and education and employment domains (N = 42).

Question		Fully completed: n (%)
Health Care		
1	Involvement in decision making process	28.0 (66.6)
2	Physician’s examination without parents	17.0 (40.4)
3	Familiarization with general practitioner	30.0 (71.4)
4	Familiarization with neurologist	25.0 (59.5)
5	Familiarization with pulmonologist	27.0 (64.2)
6	Familiarization with cardiologist	23.0 (54.7)
7	Familiarization with others care providers	23.0 (54.7)
8	Familiarization with endocrinologist	19.0 (45.2)
9	Familiarization with social worker	17.0 (40.4)
10	Medical record transfer to adult care	16.0 (38.0)
11	Access to medical information	27.0 (64.2)
12	Conversations on medical findings	24.0 (57.1)
13	Conversations on anticipated changes in health	29.0 (69.0)
14	Conversations on anticipated changes in privacy	24.0 (57.1)
15	Determining the need in decision-making support	26.0 (61.9)
16	Consultation regarding palliative care	14.0 (33.3)
*Average percentage*		*54*.*8*
**Education and Employment**		
1	Evaluating studies opportunities	18.0 (42.8)
2	Familiarization with strengths and interests	28.0 (66.6)
3	Evaluate clashes between medical needs and studies/work	25.0 (59.5)
4	sources needed for education/employment -familiarization	25.0 (59.5)
5	Education/employment support—Social Security Services	18.0 (42.8)
6	Approaching education/employment support groups	10.0 (23.8)
7	Approaching the university concerning medical needs	20.0 (47.6)
8	Initiation of planning living options while studying	18.0 (42.8)
9	Initiation of planning living options after studying	17.0 (40.4)
*Average percentage*		*47*.*3*

**Table 3:** Transition readiness: Housing & transportation, activities of daily living, independent life & autonomy.

Question		Fully completed: n (%)
**Housing and Transportation**		
1	Discussing possible adult living arrangements	20.0 (47.6)
2	Performing family home accessibility check	33.0 (78.5)
3	Evaluating other possible accessible living arrangements	21.0 (50.0)
4	Accessibility experts’ consultation–required changes	19.0 (45.2)
5	Social worker consultation–housing assistance eligibility	10.0 (23.8)
6	Discussing mobility with the family car	34.0 (80.9)
7	Evaluating mobility options–public transportation	21.0 (50.0)
8	Evaluating independent driving option	25.0 (59.5)
*Average percentage*		*54*.*4*
**Activities of Daily living**		
1	Receiving instruction–hiring aid	15.0 (35.7)
2	Receiving consultation—accessories/ assistive technology	15.0 (35.7)
3	Receiving consultation–assistive communication devices	18.0 (42.8)
4	Evaluating guardian needs in various domains	24.0 (57.1)
*Average percentage*		*42*.*8*
**Independent Life and Autonomy**		
1	Checking the ability to exercise the right to vote	38.0 (90.4)
2	Check volunteering options for the army	27.0 (64.2)
3	Carrying out actions to achieve financial independence	30.0 (71.4)
4	Investment in social relationships	30.0 (71.4)
5	Discussion of social relationships	29.0 (69.0)
6	Knowledge—activities that promote social relationships	32.0 (76.1)
7	Knowledge—contacts for social skills assistance	24.0 (57.1)
8	Knowledge—actions that promote relationships	20.0 (47.6)
9	Awareness of the ability to have sex	29.0 (69.0)
10	Knowledge—seeking intimate professional help	20.0 (47.6)
11	Receiving genetic counseling	25.0 (59.5)
12	Awareness—emotional consequences of social isolation	36.0 (85.7)
*Average percentage*		*67*.*4*

**Table 4:** Differences in transition total scores according to muscular dystrophy (N = 42).

	Muscular dystrophy type		
	Duchenne Muscular Dystrophy (N = 25): Mean (SD)	Becker Muscular Dystrophy (N = 17): Mean (SD)	t-value (p-value)
**Health Care–general**	33.1 (10.9)	40.6 (8.5)	-1.71 (0.10)
**Health Care–caregivers**	15.7 (4.7)	17.0 (4.1)	-0.86 (0.39)
**Education and Employment**	19.8 (5.7)	20.6 (6.2)	-0.37 (0.70)
**Housing and Transportation**	18.0 (4.2)	19.1 (6.1)	-0.52 (0.60)
**Activities of Daily Living**	8.5 (2.5)	9.7 (2.8)	-1.13 (0.26)
**Independent Life and Autonomy**	29.3 (5.5)	31.1 (6.2)	-0.84 (0.40)

Notes: In each domain answers were on a 3-point scale: "Need help" = 1 point, "Partially completed" = 2 points, and "Fully completed" = 3 points with higher scores indicating better transition readiness; SD, standard deviation

**Table 5:** Prediction of transition readiness.

Transition domain	Predictors	Coefficient	Standard error	t	p
**Health Care–general**	(constant)	26.67	-	-	-
	Siblings, number	2.04	0.89	2.27	**0.03**
	*Model summary*	F-ratio = 5.18; p = 0.03; Adjusted R^2^ = 0.15			
**Health Care–caregivers**	(constant)	12.09	-	-	-
	Siblings, number	0.83	0.39	2.09	**0.04**
	*Model summary*	F-ratio = 4.39; p = 0.04; Adjusted R^2^ = 0.10			
**Education and Employment**	(constant)	5.82	-	-	-
	Religiousness level: Secular/Traditional vs. religious	6.17-	2.36	2.61-	**0.01**
	*Model summary*	F-ratio = 4.14; p = 0.03; Adjusted R^2^ = 0.15			
**Housing and Transportation**	(constant)	17.11	-	-	-
	Siblings, number	-1.05	1.49	-0.70	0.50
	Religiousness level: Secular/Traditional vs. religious	-1.59	7.81	-0.20	0.84
	*Model summary*	F-ratio = 0.91; p = 0.47; Adjusted R^2^ = 0.02			
**Activities of Daily Living**	(constant)	5.49	-	-	-
	Siblings, number	0.71	0.24	2.92	**0.008**
	*Model summary*	F-ratio = 8.56; p = 0.008; Adjusted R^2^ = 0.20			
**Independent Life and Autonomy**	(constant)	Analysis was not conducted			

In the domain of health care, 45% of participants reported they need help or partially completed the various items. For 12 of the 16 items, no statistically significant differences were found based on the two task completion categories (*p* > .05). For three items (involvement in the decision-making process, familiarization with general practitioner, and conversations on anticipated changes in health), many participants reported that they fully completed the task (66.6%–71.5%). For one item (consultation regrading palliative care), fewer participants reported having fully completed the task (33.3%; Table 2).

Regarding education and employment, a small percentage reported fully completing two items: approaching employment or educational support groups (23.8%) and initiation of planning living options after studying (40.4%; Table 2).

In the domain of housing and transportation plans, one item (consultation with a social worker regarding eligibility for residential assistance), a small percentage of participants reported fully completing the task (23.8%). On the other hand, for two items (performing an assessment of the accessibility of the family home and discussing mobility using the family car), the frequency of those reporting having fully completed the task (78.5% and 80.9%, respectively) was very high (Table 3).

Regarding activities of daily living, fewer participants (35.7% for both) reported fully completing two items (receiving instruction on hiring aid and receiving consultation on assistive technology; Table 3).

In the independent life and autonomy domain, many participants reported fully completing six of the 12 items (69.0%–90.4%; Table 3).

#### Associations between transition domains

Significant positive statistical associations were found between most transition domains (*r* = .18–.94; *p* = .47–< .001; see S1 Table).

#### Associations between transition domains and demographic and functional characteristics

Associations between transition domains and age, number of siblings, mobility ability, and respiratory function were evaluated. Only the number of siblings was statistically significantly positively correlated with all transition domains (*r* = .37 for health–familiarization with caregivers to .43 for activities of daily living, *p* = .04–< .001) except for independent life and autonomy (*r* = .08, *p* = .71) and education and employment (*r* = .34, *p* = .08).

#### Differences in transition scores by muscular dystrophy type

No statistically significant differences between BMD and DMD were observed in transition scores (Table 4).

#### Transition prediction

The variables with significant relationships with different areas of transition or by group were entered into a regression model. The results indicate that the only significant predictor of transition status was number of siblings (health care–general, health care–caregivers, activities of daily living). The different regression models explained 2% (housing and transportation) to 20% (activities of daily living) of the explained variance in the different transition domains (Table 5).

## Discussion

Planning the transition from childhood to adulthood is crucial part of treatment that affects health and other important aspects of adult life among people with DMD or BMD [11]. This study examined the transition to adult life among individuals with DMD/BMD, focusing on five domains: health care, education and employment, housing and transportation, activities of daily living, and independent life and autonomy. The study findings revealed significant positive relationships between all transition domains. In other words, individuals who showed higher readiness for transition in one domain also tended to have high readiness in other domains. However, the levels of readiness varied across the five domains. The following discussion focuses on the aspects of transition identified as most challenging and factors predicting an individual’s ability to transition.

### Health care

In the domain of health care, approximately 46% of the participants indicated that they needed help or had partially completed their transition. In this context, three items were particularly challenging for many participants (more than 60%): establishing contact with a social worker about rights, transferring medical records to a specialized clinic, and seeking advice on palliative care. Knowing and using personal rights is an extremely important issue among people with disabilities. This is because individuals with disabilities are more susceptible to experiencing rights deprivation and exploitation in both the community and the workplace, compared to those without disabilities [17]. Therefore, it is important to empower people with DMD or BMD regarding their rights through familiarity with community services and self-advocacy skills.

In terms of transferring medical records to an adult clinic, a well-structured transition plan should support the treatment sequence during the transition from childhood to adulthood. The pediatric clinic should sustain treatment until the patient is well integrated into the adult clinic. In the process of transferring treatment, simplifying the authorization for transferring medical information between medical facilities is crucial. To facilitate the process of transferring medical files from pediatric to adult clinics, it is necessary to continue to investigate factors linked to difficulty in this domain [11].

Regarding palliative care, this approach significantly enhances the quality of life for patients facing incurable illnesses, irrespective of age, and provides support to their families [18]. By identifying and evaluating symptoms, addressing pain, and managing other challenges, this treatment approach focuses on preventing and alleviating suffering. Consequently, numerous nations have established national initiatives in the past decade to enhance and broaden palliative services. Nevertheless, these services often remain uncommon and insufficient to fulfill the demand. Only a small number of those who deal with incurable illnesses in the country are estimated to receive palliative care, with a minority benefiting from associated counseling and support services [19]. Given this context, assisting individuals with DMD or BMD and their families with understanding currently available options concerning this matter is of utmost importance.

### Education and employment

In the realm of education and employment, 74% of participants expressed a need for assistance in reaching out to support and guidance groups. Adults diagnosed with DMD or BMD can participate in meaningful occupations through various avenues. Education plays a pivotal role in enabling these meaningful occupations. Additional factors that facilitate these endeavors include support systems, accommodations, self-care proficiency, and coping strategies. In contrast, numerous occupational barriers exist, such as social expectations of a normative adulthood, discrimination and inaccessible environments, lack of support and resources, medical challenges, fatigue, lack of motivation, and social isolation and depression [20]. Support groups hold particular significance because they can assist young adults in navigating the challenges of education and occupation while facilitating connections to the appropriate resources in these areas.

### Housing and transportation

In the domain of housing and transpiration, a high percentage of participants (more than 60%) reported needing help with consulting with a social worker about eligibility for housing assistance. Similar to young people without disabilities, young people with disabilities also want to leave their parents’ home and live independently. Nonetheless, young adults with disabilities who pursue independent living confront a distinct set of challenges that extend beyond what their counterparts without disabilities experience. For example, they face issues related to physical accessibility, training in using assistive technologies in their home, and the availability of emergency services [17].

### Activities of daily living

The domain with the highest percentage of participants who reported needing help or only partially completing tasks (58% on average) was activities of daily living. These results are not surprising, given that with DMD or BMD progression, individuals experience significant physical disabilities that necessitate reliance on others for activities of daily living. However, this dependence does not preclude personal autonomy. Autonomy involves self-determination and the ability to make life choices, which can be maintained even when physical independence is compromised. Reflections on the autonomy of people with severe disabilities are usually accompanied by ambivalence. Specifically, a higher degree of dependence is usually perceived as reflecting a lower degree of personal autonomy. This ambivalence is also enhanced by some definitions of autonomy that place it in opposition to dependence [21]. Although severe physical disabilities associated with DMD or BMD reduce physical independence, they do not eliminate the capacity for autonomy. By fostering supportive relationships and using assistive technologies, individuals can exercise self-determination and maintain control over their life. Therefore, assessment of needs in the transition to adulthood is also of relevance for people with severe disabilities, such as DMD and BMD [22, 23].

In this domain, a high percentage of participants with transition difficulties (more than 60%) was found for two of four items. The first item is receiving instruction regarding hiring an aide. The employment of an aide as a caregiver, especially a foreign worker, is a complex process that includes many steps, such as obtaining an employment permit from the Population and Immigration Authority and registering with a private bureau authorized to bring in these workers for the purpose of placement [24]. The complexity of hiring an aide persists even after obtaining the necessary permits, primarily due to the limited availability of such aides. Accordingly, providing a comprehensive briefing on this subject to people with DMD or BMD and their families is of great importance.

The second item involved receiving consultation on accessories and assistive technology. Finding and matching accessories and assistive technology to personal needs is a complex task that includes characterizing the functional problem, receiving a professional opinion on the available relevant accessory or assistive technology, and learning to use the new technology [25]. It is also important to highlight that the use of assistive technologies can significantly enhance meaningful participation of individuals with severe disabilities. For example, using powered wheelchairs can greatly facilitate the development of social skills by enhancing mobility and social independence. Powered wheelchairs also provide opportunities for participation in wheelchair sports, which can enhance self-confidence and social participation and lead to further education and potential employment [26]. Hence, it is very important in the transition phase to provide advice on relevant accessories and assistive technology. Nonetheless, it is important to remember that although technology can improve mobility and independence, other factors such as environmental barriers and societal attitudes may also influence overall participation levels [27].

### Independent life and autonomy

The domain with the lowest degree of difficulty was independent life and autonomy. However, several challenging aspects of this domain emerged—namely, knowledge on actions to promote relationships and knowledge of to whom to turn for professional help regarding intimacy. Relationships and intimacy are fundamental and innate human needs. Individuals with physical disabilities often find it difficult to meet these needs because of factors such as impaired mobility and lack of knowledge about intimacy and sexual health. Specifically, people with physical disabilities are often seen as asexual, and intimacy and sexuality in relationships is often not considered a concern in this population. These misconceptions can result in individuals with physical disabilities not receiving guidance when issues arise. Not receiving this attention may impede people with disabilities from forming relationships and intimacy. Therefore, efforts should be made to provide more inclusive education on life skills for people with DMD or BMD. Providing such education will better meet the basic human needs of an often underserved and stigmatized population [28].

### Transition prediction

Regarding transition scores, two significant predictors were found: age and number of brothers and sisters. Age significantly predicted status in only one transition domain, education and employment. The other null results related to age were not surprising, given that although adult role attainment is expected to increase with age, the progressive nature of DMD or BMD leads to worse motor function over time [3], which may influence the ability to attain adult responsibilities. Similarly, among individuals with DMD, the ability to manage a medication regimen has been shown to improve with increasing age, alongside a trend toward worse arm function [6]. This finding reinforces the argument that although a structured transition process should begin years before the transition, support for the transition process should be flexible and not fixed by biological age [29].

The reported association between arm function and ability to manage medications [6] contrasts with the null association found in the current study between functional level and transition ability. Function in the current study was assessed using ordinal scales, with most participants fully using assistive devices for ambulation (67%) and remaining independent in respiratory function (62%). The ordinal nature of these scales and the relatively limited between-participant variability in functional level may be responsible for the observed lack of association between functional level and transition. Future studies should use continuous scales to measure function (e.g., grip strength test).

The number of siblings significantly predicted several transition domains scores. More precisely, the greater the number of siblings, the higher the transition score. Numerous studies have evaluated the emotional impact of having a sibling with a disability. However, the impact of having siblings on children with a disability has been rarely addressed in the literature. Moreover, to the best of our knowledge, the extent to which having siblings enhances the transition to adulthood among young people with DMD or BMD has never been examined. Although the relationship with siblings usually does not function as a secure base in the same way as the parental relationship, it may represent an important source of emotional support and contribute to the self-concept of the adolescent [30]. Self-concept is an individual’s self-beliefs, including personal attributes and self-identity. Having a high self-concept is critical to the transition process of people with a disability and their family. Specifically, high self-concept may give the young person courage in the transition phase. Having siblings may also enhance their sense that they have someone who listens to them, pays attention to their health needs, and more. These factors may give the person with the disability more courage to embark on the transition to adulthood [31].

Dystrophy type (DMD vs. BMD) did not predict transition readiness. These results were surprising, because DMD disease severity is typically greater than that of BMD. These surprising results can be partly explained by functional adaptations and medical support that mitigate disease impact. More specifically, individuals with DMD reported widespread use of full-time mobility devices (84%), which can enhance their independence and ability to participate in daily life. These devices compensate for the severe loss of muscle strength and allow patients to maintain some degree of autonomy, enabling them to engage in education, social activities, and other life domains essential for transition readiness. In contrast, a lower percentage of participants with BMD (37%) used mobility devices, reflecting their generally milder symptoms but potentially leading to varied mobility-related challenges.

Moreover, despite the progressive nature of DMD, 60% of individuals with DMD in the current study said they could breathe independently or used partial noninvasive respiratory support, compared to 100% in the BMD group. This relatively high level of respiratory independence among participants with DMD likely reflects advances in medical care and management, which enable them to maintain functional capabilities critical for transitioning to adulthood. Therefore, it appears that the availability of assistive technologies and partial respiratory support in DMD may reduce the disparity in disease burden between these two groups. These interventions likely play a significant role in empowering DMD patients to achieve levels of independence, self-care, and social participation comparable to their peers with BMD, facilitating a smoother transition to adult life.

Finally, the null differences in transition between the two muscular dystrophy groups may suggest that they face common challenges, such as navigating health care systems, addressing educational and vocational needs, and overcoming societal and accessibility barriers. The similarity in transition readiness could reflect shared experiences and resources, rather than disease severity alone.

### Clinical implications

This study provided valuable insights for developing interventions to support individuals with DMD and BMD in their transition to adulthood. The findings highlight the interconnected nature of transition readiness across domains, underscoring that improvements in one area often correlate with enhanced readiness in others. Therefore, interventions should adopt a holistic approach, addressing multiple domains simultaneously. For example, empowering individuals in the domain of health care by improving their ability to navigate systems and access resources may also bolster their independence in daily living and other areas of life.

Challenges identified in this study, such as transferring medical records, understanding legal rights, and seeking palliative care, suggest a need for structured transition programs. These programs should include training on navigating health care systems, advocacy for rights, and support for emotional and practical aspects of end-of-life care planning. Education and employment interventions should emphasize access to support groups, skill-building programs, and accommodations that address physical, social, and systemic barriers. Similarly, addressing challenges in housing and transportation requires a focus on physical accessibility, availability of adaptive technologies, and financial support for independent living. The study also highlighted the importance of assistive technologies in enabling independence and participation, particularly in the domain of activities of daily living. Providing individuals and families with guidance on selecting, acquiring, and using these technologies will be crucial. Last, concerns about fostering relationships and addressing intimacy in the domain of autonomy reflect the need for inclusive education and counseling services. These interventions should work to combat stigma surrounding disability and sexuality, ensuring that individuals with DMD or BMD can develop fulfilling personal relationships.

### Limitations

Our study also has some limitations. First, interpretation of the study findings is limited by the small sample size. Despite this limitation, it is important to note that the data were drawn from a population-based registrar system and not from a specific clinic catering to individuals with DMD or BMD. Additionally, it is worth noting that there are only 300 DMD and BMD patients in Israel, and the study enrolled only patients who were 16–35 years old. Consequently, although the sample size was relatively small, it may still be representative of the DMD and BMD population in the country. Second, we did not ask participants if a caregiver helped them complete the survey, which may have biased the responses. In addition, the generalizability of the study is limited to youth with the cognitive ability needed to complete the questionnaire. Finally, on account of the small sample size, we did not examine the influence of several important clinical factors, such as mutation type, on transition ability. We also did not examine the influence of religiosity level on transition status. It is important to investigate this topic, because individuals with varying levels of religiosity often differ in numerous sociodemographic characteristics (e.g., education) and lifestyles, which may influence their transition readiness and outcomes.

## Conclusions

In conclusion, the study findings reinforce the impact of DMD and BMD on the transition to adult roles. More specifically, the survey data suggest that activities of daily living—mainly hiring an aide and consulting on accessories and assistive technology—is an important area of need. Another domain with a high percentage of participants needing help was education and employment, especially in approaching support groups and initiating planning of living options after education.

The domain with the lowest degree of difficulty was independent life and autonomy. Nonetheless, several challenging aspects were identified in this domain—namely, knowledge on actions to promote relationships and knowledge of to whom to turn for professional help regarding intimacy. In addition, the lack of a connection between age and most domains of transition points to the need for long-term accompaniment of the young person regardless of biological age.

The current study also yielded some unique findings, such as the null association of the level of motor and respiratory disability on transition outcomes, and the significance of sociodemographic factors like the number of siblings. These results may be relevant for professionals aiming to develop training programs to facilitate the transition of people with DMD or BMD to new adult roles and responsibilities.

## Supporting information

S1 Table Associations (Spearman’s rho correlations) between transition domains (N = 42). (DOC)

S1 Data De-identified data files used for statistical analyses. (XLS)
