# Supplementary material for: Navigating adulthood: Exploring the transition needs of adolescents and young adults affected by Duchenne or Becker muscular dystrophy
Source: PLoS One. 2025 Jan 14;20(1):e0317006. doi: 10.1371/journal.pone.0317006 (PMC11731756; doi:10.1371/journal.pone.0317006)
Supplement: S1 Table — (DOC) [file pone.0317006.s001.doc]

**Supporting table 1.**

Associations (Spearman's rho correlations) between transition domains (N=42)

| **Independent Life and Autonomy** | **Activities of Daily Living** | **Housing and Transportation** | **Education and Employment** | **Health Care- familiarization with givers** | **Health Care - general** |  |
| --- | --- | --- | --- | --- | --- | --- |
| - | - | - | - | - | - | **Health Care - general** |
| - | - | - | - | - | 0.94 (<0.001) | **Health Care-familiarization with caregivers** |
| - | - | - | - | 0.35 (0.04) | 0.38 (0.08) | **Education and Employment** |
| - | - | - | 0.87 (<0.001) | 0.45 (0.01) | 0.54 (0.01) | **Housing and Transportation** |
| - | - | 0.65 (<0.001) | 0.66 (<0.001) | 0.65 (<0.001) | 0.62 (0.007) | **Activities of Daily Living** |
| - | 0.48 (0.01) | 0.59 (0.001) | 0.56 (0.001) | 0.24 (0.20) | 0-.18 (0.47) | **Independent Life and Autonomy** |
